# Correction: Versatile and automated workflow for the analysis of oligodendroglial calcium signals

**DOI:** 10.1007/s00018-024-05171-w

**Published:** 2024-03-01

**Authors:** Dorien A. Maas, Blandine Manot-Saillet, Philippe Bun, Chloé Habermacher, Corinne Poilbout, Filippo Rusconi, Maria Cecilia Angulo

**Affiliations:** 1 Université Paris Cité, Institute of Psychiatry and Neuroscience of Paris (IPNP), INSERM U1266, “Team: Interactions Between Neurons and Oligodendroglia in Myelination and Myelin Repair”, 75014 Paris, France; 2 Université Paris Cité, Institute of Psychiatry and Neuroscience of Paris (IPNP), INSERM U1266, “NeurImag Platform”, 75014 Paris, France; 3 GHU PARIS Psychiatrie and Neurosciences, 75014 Paris, France; 4 IDEEV, GQE, Université Paris-Saclay, CNRS, INRAE, AgroParisTech, 12, Route 128, 91272 Gif-sur-Yvette, France; 5 Centre de Recherche des Cordeliers, Sorbonne Université, INSERM, Université Paris Cité, 75006 Paris, France; 6 Present Address: SynapCell, Bâtiment Synergy Zac Isiparc, 38330 Saint Ismier, France

**Correction: Cellular and Molecular Life Sciences (2024) 81:15** 10.1007/s00018-023-05065-3

In this article Figs. 1, 3 and 6 were corrupted in the article PDF; the figures should have appeared as shown below.

**Fig. 1:**
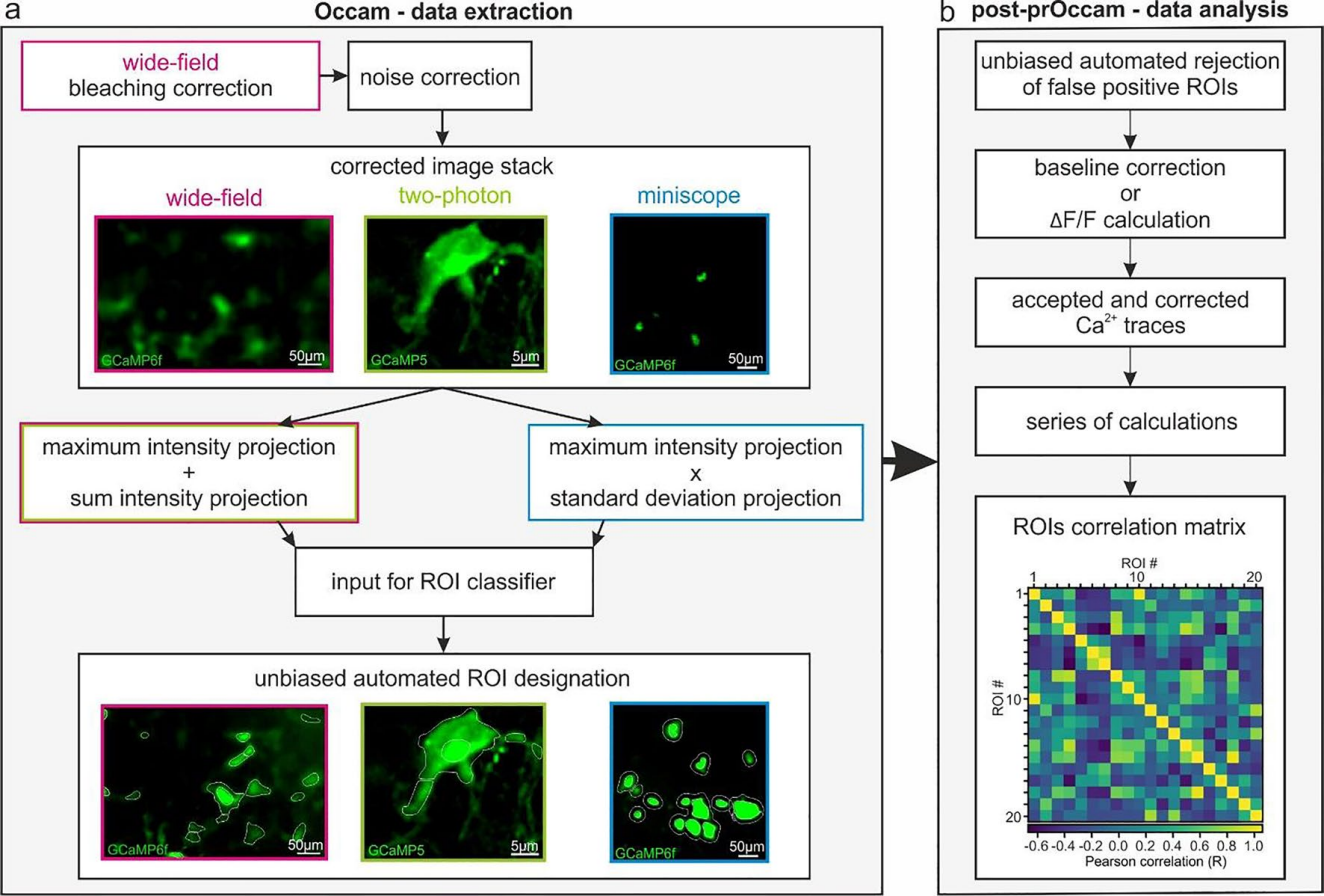
Occam and post-prOccam: an automated analysis software solution for oligodendroglia Ca^2+^ imaging of different preparations. **a** The Occam software is available as a Fiji/ImageJ2 plugin and configurable for the analysis of wide-field, two-photon and miniscope Ca^2+^ imaging. Occam performs bleaching correction only on wide-field image stacks and does noise correction according to the imaging condition (Supplementary manual). Then, it uses the maximum and sum intensity projections for wide-field and two-photon image stacks and the maximum and standard deviation projections for miniscope image stacks to build a projection image used as input for the WEKA-based ROI classifier. **b** Output from Occam is fed to the post-prOccam Python-based software that (1) rejects any ROI that does not show significant Ca^2+^ fluctuations; (2) performs either baseline subtraction or the conventional Δ*F*/*F* correction; (3) performs statistical calculations for each accepted ROI; and (4) computes a ROIs Pearson correlation matrix. Occam and post-prOccam are multiplatform, free and open source programs, freely available at: https://gitlab.com/d5674/occam (detailed procedures and software inner workings are described in the Supplementary manual)

**Fig. 3:**
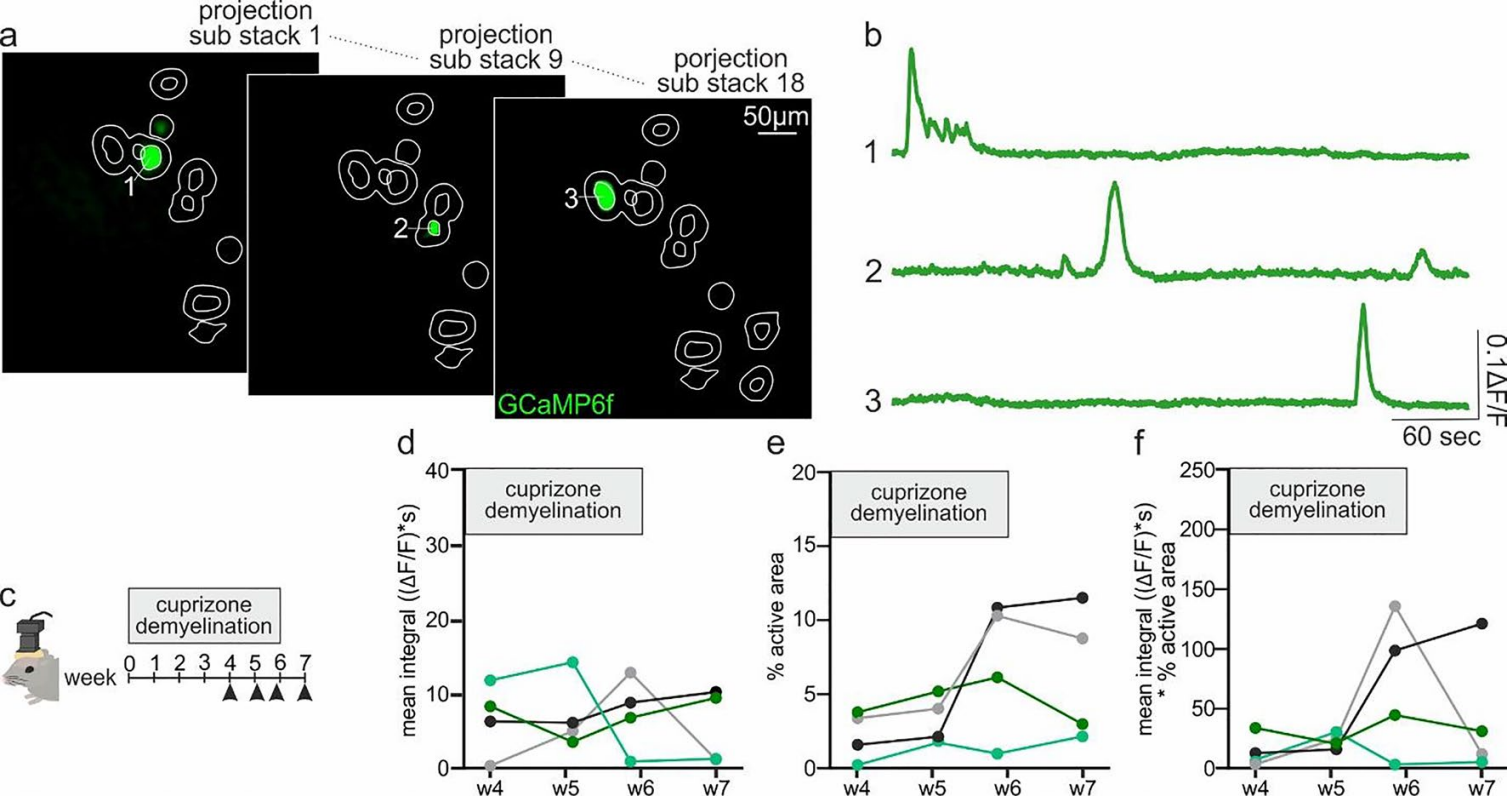
Analysis of miniscope Ca^2+^ signals of oligodendroglia in freely moving mice. **a** Representative images of an in vivo microendoscopy Ca^2+^ imaging stack collected from a demyelinated corpus callosum of a freely moving mouse. The image displays detected active ROIs (white) in several sub-stacks as obtained with the in vivo analysis option of Occam and **b** their corresponding corrected Ca^2+^ traces obtained with post-prOccam. **c** Longitudinal experiments that can be analyzed with Occam and post-prOccam over weeks. Four mice were fed with the cuprizone diet to induce demyelination (see Supplementary Material and Methods) and Ca^2+^ imaging was performed for four consecutive weeks. **d** Mean integral, **e** % of active area and **f** mean integral multiplied by percentage of active area are calculated with post-prOccam over the four consecutive weeks for all mice

**Fig. 6:**
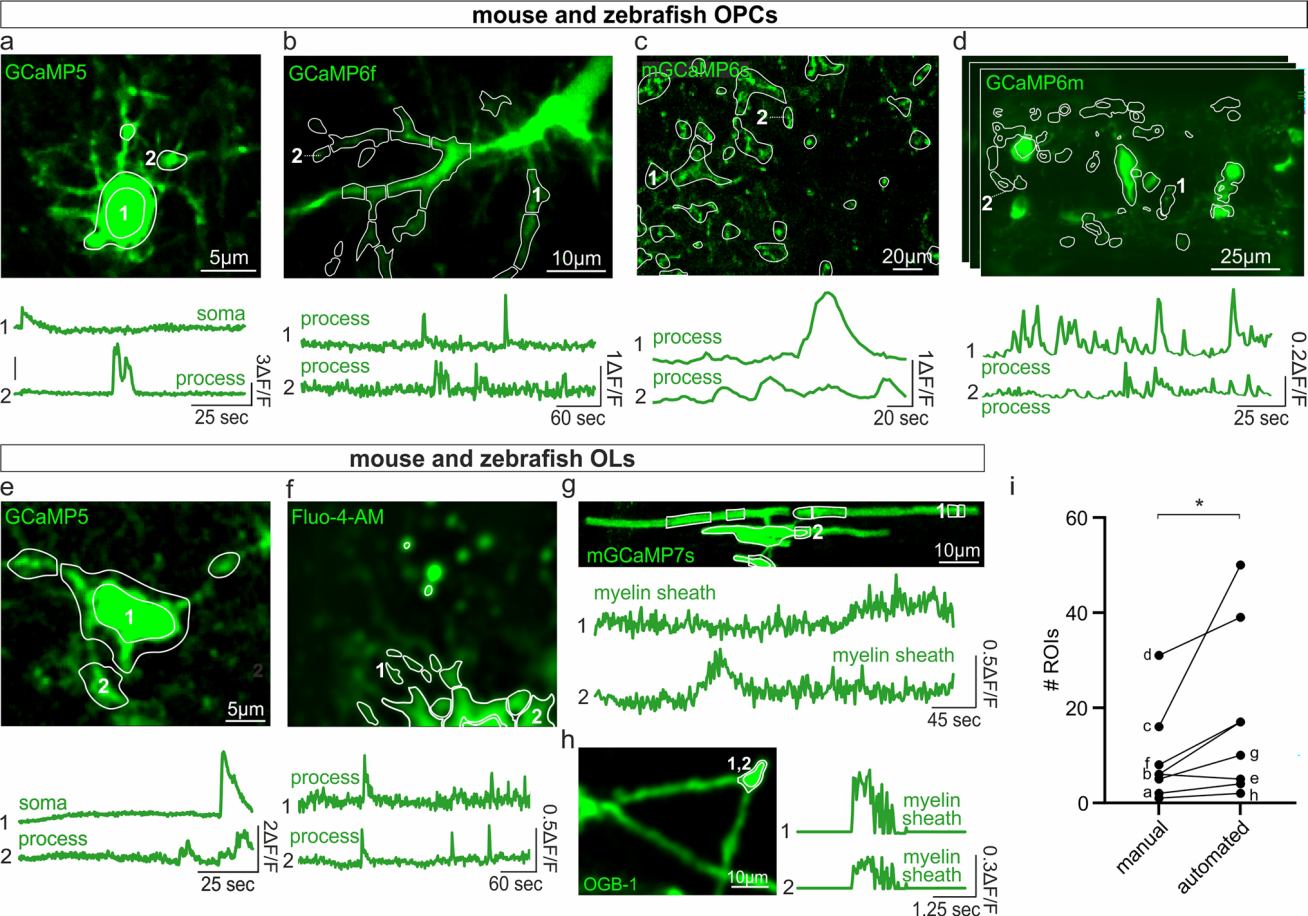
Occam and post-prOccam analyze Ca^2+^ signals from OPCs, OLs and myelin sheaths in different species and imaging conditions. Representative images with ROIs (white) and Ca^2+^ traces obtained with Occam and post-prOccam for (**a**) a putative OPC from mouse demyelinated corpus callosum in acute slices recorded in a two-photon microscope (see “Materials and methods”), (**b**) an OPC from the in vivo mouse somatosensory cortex recorded with a two-photon microscope (Supplementary Video 1 from [11], (**c**) OPC processes from the in vivo mouse visual cortex recorded with a two-photon microscope (Supplementary Video 3 from [18], (**d**) OPC somata and processes from the in vivo zebrafish spinal cord recorded with a light sheet microscope (from Tim Czopka & Patricia Bishop, Unpublished data), (**e**) a putative OL from mouse demyelinated corpus callosum in acute slices recorded with a two-photon microscope (see “Materials and methods”), (**f**) a primary mouse OL in culture recorded with an Opterra II Multipoint Swept Field Confocal microscope (Supplementary Video 1 from [14], (**g**) an OL process from the in vivo zebrafish spinal cord recorded with a Confocal Zeiss LSM880 Airyscan (from Philipp Braaker and David Lyons, unpublished data) and (**h**) an OL process recorded in an acute brain slice from a mouse with an Olympus BX61WI microscope and a NeuroCCD camera at 40 Hz (Supplementary Video 1 from [6]. The image stacks were recorded at different acquisition rates and analyzed with Occam using either two-photon (**a**, **b**, **c**, **f**, **g**) or miniscope configuration (**d**). **i** Comparison of the number of ROIs identified by visual inspection with that of ROIs automatically detected in the different analyzed stacks. *p < 0.05; Wilcoxon rank test. Dot plots are presented as mean ± s.e.m

The original article has been corrected.

